# Bilharziose vulvaire chez une fille de 10 ans à Kirane-Mali: rapport de cas

**DOI:** 10.11604/pamj.2021.39.2.28693

**Published:** 2021-05-03

**Authors:** Bakary Sayon Kéita, Tiémoko Sogodogo, Drissa Goïta, Ousmane Diawara, Djibril Doucouré, Madou Traoré, Sidy Sangaré, Mamadou Sayon Kéita, Manifa Théra, Sounkalo Dao

**Affiliations:** 1 Service de Médecine et Spécialités Médicales/Unité d'Infectiologie, l'Hôpital Fousseyni Daou, Kayes, Mali,; 2 Cabinet Médical “Kaniaga”, Kirané, Mali,; 3 Service de Médecine, Hôpital de Sikasso, Sikasso, Mali,; 4 Centre de Santé Communautaire de Kayes N'Di/Kayes, Kayes, Mali,; 5 Service de Chirurgie et Spécialités Chirurgicales de l'Hôpital Fousseyni Daou, Kayes, Mali,; 6 Cellule Sectorielle de Lutte Contre le VIH/Sida, la Tuberculose et les Hépatites Virales du Ministère de la Santé et des Affaires Sociales (CSLS-TBH/MSAS), Bamako, Mali,; 7 Direction Régionale de la Santé/Division Planification, Kayes, Mali,; 8 Service des Maladies Infectieuses du CHU du Point G, Bamako, Mali

**Keywords:** Schistosomiase, biopsie, praziquantel, Mali, rapport de cas, Schistosomiasis, biopsy, praziquantel, Mali, case report

## Abstract

La bilharziose génitale chez la femme se trouve principalement dans le col de l'utérus et le vagin, moins fréquemment sur la vulve et dans les trompes de Fallope et les ovaires, et rarement dans le corps de l'utérus. Nous rapportons le cas d´une fille, âgée de 10 ans, admise pour tuméfaction vulvaire, chez qui l'histologie a conclu à une bilharziose cutanée à Schistosoma haematobium. L'évolution a été favorable sous praziquantel 40 mg/kg en une prise unique avec régression de la tumeur.

## Abstract (en)

Genital schistosomiasis is mainly located in the neck of the uterus and the vagina, less frequently on the vulva, the fallopian tubes and ovaries and rarely in the body of the uterus. We here report the case of a 10-year-old girl admitted with a swelling on the vulva in whom histological examination showed cutaneous schistosomiasis due to Schistosoma haematobium. Outcome was favorable with a single 40 mg/kg mg dose of praziquantel, with tumor regression.

## Introduction

La schistosomiase reste un problème de santé publique important qui nuit au développement social et économique des régions tropicales du monde, principalement en Afrique sub-Saharienne [1]. La bilharziose génitale féminine, due essentiellement à l´espèce *Schistosoma haematobium*, reste une entité rarement décrite [2]. Elles représentent 1/4 des schistosomoses diagnostiquées par l´histologie, sur le col utérin, la trompe, le vagin et l´ovaire [3]. Nous décrivons 1 cas de bilharziose vulvaire chez une fille de 10 ans diagnostiquée par l´histologie.

## Patient et observation

Il s´agit d´une fille de 10 ans originaire d´Asseyif en République Islamique de Mauritanie qui a été admise en consultation pour une masse vulvaire. L´anamnèse a mis en évidence son appartenance à l´ethnie nomade peulh. L´anamnèse a également révélé que la masse vulvaire était de la taille d´un point trois mois auparavant et avait progressivement augmenté de taille jusqu´à la taille actuelle. Il n´y avait aucune plainte à type de douleur locale, de prurit, de brulure en dehors de l´esthétique.

**Figure 1:**
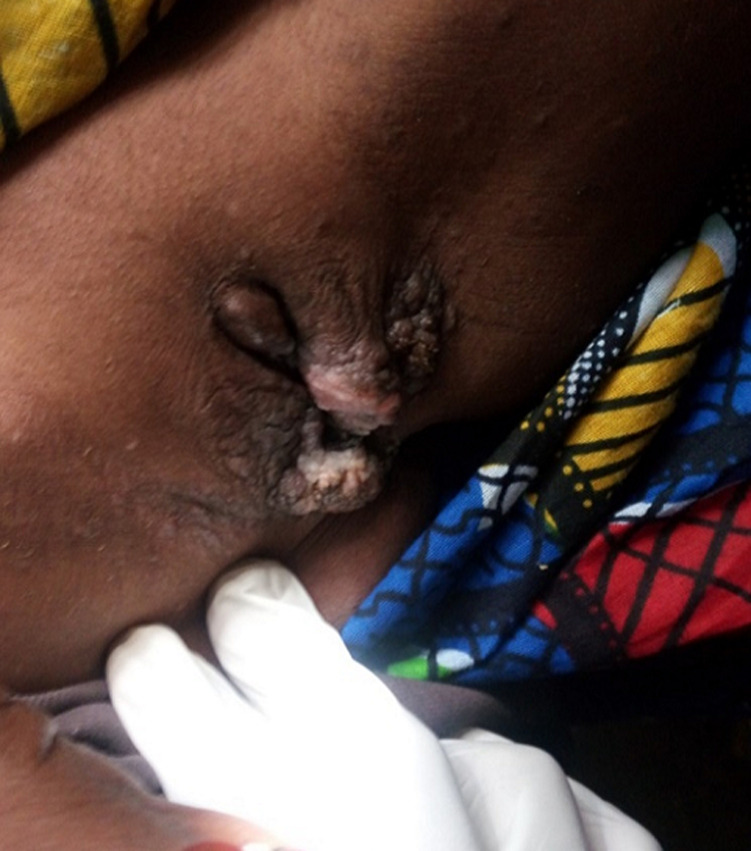
tumeur vulvaire, volumineuse, irrégulière et squameuse envahissant les deux grandes lèvres et une partie des petites lèvres confirmée à l'histologie comme bilharziose vulvaire chez une fillete de 10 ans vue en consultation à Kirané (Mali)

**Figure 2:**
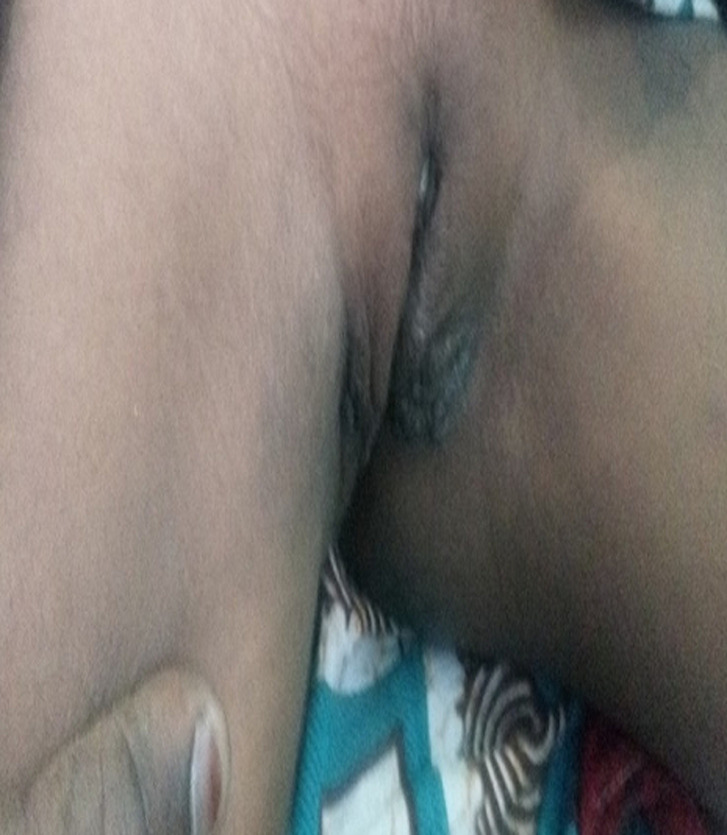
régression deux semaines après la prise de praziquantel de la tumeur vulvaire décrite en figure 1 et qui a été confirmée à l'histologie comme bilharziose vulvaire

L´examen des organes génitaux externes met en évidence une tumeur vulvaire, volumineuse, irrégulière et squameuse envahissant les 2 grandes lèvres et une partie des petites lèvres (Figure 1). La cavité vaginale était rétrécie, le méat urétral visible et perméable, la marge annale et le périnée étaient d´aspect normal. Par ailleurs on notait des adénopathies inguinales bilatérales et sensibles, la température à 37.8°C, le poids à 29kg et le reste de l´examen physique était sans particularité. Une biopsie a pu être réalisée. L´examen anatomo-pathologique a conclu à une bilharziose cutanée à *Schistosoma haematobium*. La patiente a été traitée par le Biltricide (praziquantel) 600mg en raison de 40mg/kg poids en comprimé et en prise unique. Deux semaines après la prise de praziquantel la patiente a été reçue en consultation et nous avions noté une bonne évolution clinique avec régression de la tumeur (Figure 2).

## Discussion

La schistosomiase reste un problème de santé publique important qui nuit au développement social et économique des régions tropicales du monde, principalement en Afrique sub-Saharienne [2]. Au Mali, les enquêtes épidémiologiques réalisées par le Programme National de Lutte contre les Schistosomoses montrent que la totalité du pays est touchée par cette affection [4]. La localisation de l´infection bilharzienne dans le tractus génital est retrouvée chez 75% des femmes présentant la bilharziose urinaire. Pourtant elle est rarement symptomatique et les publications consistent surtout à des rapports des cas [5]. D´où la présente observation. L´âge de notre patiente était de 10 ans, proche de la moyenne d´âge de la série de Faye O *et al*. portant sur 8 cas de schistosomose vulvaire dans laquelle l´âge moyen était de 11 ans (extrêmes de 7 à 13 ans) [6], inférieur à celui du cas rapporté par Bourée P *et al*. [2] qui était de 25 ans. La localisation vulvaire est retrouvée aussi bien chez les fillettes que chez les femmes d´âge mur.

Chez notre cas rapporté il s´agissait d´une tumeur vulvaire, volumineuse, irrégulière et squameuse envahissant les 2 grandes lèvres et une partie des petites lèvres par contre dans la série de Faye O *et al*. il s´agissait de nodules végétants et/ou ulcérés (6 cas), de papule isolée (1 cas) d´ulcération sur fond infiltré (1 cas). Toutes les lésions étaient localisées sur le versant gauche de la vulve (lèvres) ou l´aine homolatérale [6]. Il n´existe pas de signes cliniques spécifiques de la bilharziose génitale. Si des arguments épidémiologiques existent, il faut penser aussi à la bilharziose génitale devant: une bilharziose urinaire ou intestinale; une leucorrhée résistant aux traitements habituels anti-infectieux ou antimycosiques; des douleurs pelviennes chroniques avec ou sans troubles du cycle menstruel; des lésions cervicales et/ou vaginales à type: de tapis sableux, de leucoplasie, de nodules, d´ulcération ou de bourgeon; une tumeur vulvaire ou ovarienne; une stérilité tubaire [7].

Dans notre cas rapporté c´est l´examen anatomo-pathologique qui a permis de posé le diagnostic. Le diagnostic de certitude de la bilharziose génitale est avant tout histologique [3]. Nous avions noté une bonne évolution clinique avec régression de la tumeur chez notre patiente deux semaines après la prise de Biltricide (praziquantel) 600mg en raison de 40mg/kg poids en comprimé et en prise unique. Dans le cas rapporté par Fall PA *et al*. le traitement a constitué en une exérèse de la tumeur suivie de deux cures de praziquantel, à 15 jours d´intervalle [8]. L´affaissement et la disparition des lésions peuvent varier de quelques semaines à plusieurs mois. Pour les lésions nodulaires de grande taille, le traitement médical doit toujours être suivi d´une exérèse chirurgicale des lésions résiduelles [6].

## Conclusion

Les lésions bilharziennes vulvaires peuvent simuler une tumeur maligne. Seul l´analyse anatomopathologique permet de poser un diagnostic de certitude et dans notre cas le praziquantel en prise unique s´est montré efficace.
